# Fetal weight estimation at term – ultrasound versus clinical examination with Leopold’s manoeuvres: a prospective blinded observational study

**DOI:** 10.1186/s12884-019-2251-5

**Published:** 2019-04-11

**Authors:** Oliver Preyer, Heinrich Husslein, Nicole Concin, Anna Ridder, Maciej Musielak, Christian Pfeifer, Willi Oberaigner, Peter Husslein

**Affiliations:** 1 Department of Obstetrics and Gynaecology, University Teaching Hospital Tauernklinikum Zell am See, Paracelsusstrasse 8, A-5700 Zell am See, Austria; 2 Department of Obstetrics and Gynaecology, Division of General Gynaecology and Gynaecologic Oncology, Medical University of Vienna, Waehringer Guertel 18-20, A-1090 Vienna, Austria; 3 Department of Obstetrics and Gynaecology, Medical University of Innsbruck, Anichstrasse 35, A-6020 Innsbruck, Austria; 4 Paracelsus Medical University, Strubergasse 21, A-5020 Salzburg, Austria; 5 Department of Clinical Epidemiology of the Tyrolean State Hospitals Ltd, Cancer Registry of Tyrol, Tirolkliniken GmbH, Anichstrasse 35, A-6020 Innsbruck, Austria; 6 Department of Public Health, Health Services Research and Health Technology Assessment, Institute of Public Health, Medical Decision Making and HTA, UMIT The Health & Life Sciences University, Eduard-Wallnöfer-Zentrum 1, A-6060 Hall in Tirol, Austria; 7 Department of Obstetrics and Gynaecology, Division of Obstetrics and Fetomaternal Medicine, Medical University of Vienna, Waehringer Guertel 18-20, A-1090 Vienna, Austria

**Keywords:** Prospective blinded observational study, Ultrasound, Estimated fetal weight, Body mass index, Clinical examination, Fetal weight estimation, Leopold’s manoeuvres, Normal weight, Overweight

## Abstract

**Background:**

Fetal weight estimation is of key importance in the decision-making process for obstetric planning and management. The literature is inconsistent on the accuracy of measurements with either ultrasound or clinical examination, known as Leopold’s manoeuvres, shortly before term. Maternal BMI is a confounding factor because it is associated with both the fetal weight and the accuracy of fetal weight estimation. The aim of our study was to compare the accuracy of fetal weight estimation performed with ultrasound and with clinical examination with respect to BMI.

**Methods:**

In this prospective blinded observational study we investigated the accuracy of clinical examination as compared to ultrasound measurement in fetal weight estimation, taking the actual birth weight as the gold standard.

In a cohort of all consecutive patients who presented in our department from January 2016 to May 2017 to register for delivery at ≥37 weeks, examination was done by ultrasound and Leopold’s manoeuvres to estimate fetal weight. All examiners (midwives and physicians) had about the same level of professional experience.

The primary aim was to compare overall absolute error, overall absolute percent error, absolute percent error > 10% and absolute percent error > 20% for weight estimation by ultrasound and by means of Leopold’s manoeuvres versus the actual birth weight as the given gold standard, namely separately for normal weight and for overweight pregnant women.

**Results:**

Five hundred forty-three patients were included in the data analysis. The accuracy of fetal weight estimation was significantly better with ultrasound than with Leopold’s manoeuvres in all absolute error calculations made in overweight pregnant women. For all error calculations performed in normal weight pregnant women, no statistically significant difference was seen in the accuracy of fetal weight estimation between ultrasound and Leopold’s manoeuvres.

**Conclusions:**

Data from our prospective blinded observational study show a significantly better accuracy of ultrasound for fetal weight estimation in overweight pregnant women only as compared to Leopold’s manoeuvres with a significant difference in absolute error. We did not observe significantly better accuracy of ultrasound as compared to Leopold’s manoeuvres in normal weight women. Further research is needed to analyse the situation in normal weight women.

## Background

Accuracy of fetal weight estimation is of key importance in antenatal care, as well as in the planning and management of labour and mode of delivery [1–9].

In order to achieve more accurate prenatal fetal weight estimations and align these with a risk-optimizing mode of delivery, additional tools supporting the standard of use with ultrasound are needed.

The main ultrasonic methods used to calculate the weight of a fetus are based on measurement of fetal abdominal circumference (AC) and estimated fetal weight (EFW) using a formula first described by Hadlock et al. [10, 11], and the sufficient accuracy of this model has recently been proven [12].

Antenatal magnetic resonance imaging (MRI) [13] or soft-tissue measurements [14] have been shown to be of no benefit in improving the accuracy of fetal weight estimation.

Leopold’s manoeuvres have a long-standing tradition in obstetrics and midwifery and were first described by the German gynaecologist Christian Gerhard Leopold (1846–1911) in the journal “Archiv für Gynäkologie” in the 19th century [15]. By placing both hands on the woman’s abdomen the examiner can describe the position of the fetus as well as the level of the uterine fundus and thus detect a disproportion between fetus and the female pelvis. Experienced examiners are able to give a clinical estimation of fetal weight after performing Leopold’s manoeuvres including symphysis-fundal height and abdominal palpation [1].

Maternal body mass index (BMI) has been shown to affect the accuracy of EFW [16]. Clinicians should be aware of the limitation of sonographic fetal weight estimation, especially in obese patients, as maternal body mass index influences sonographic fetal weight estimation prior to scheduled delivery and the measurement deviation is greater in pregnant women with a BMI ≥ 25 [17–19].

We examined whether clinical assessment is an alternative when ultrasound is not available or can serve as a useful supplemental examination using the actual birth weight as the gold standard. The aim of our prospective blinded observational study was to evaluate the accuracy of fetal weight estimation performed with ultrasound and clinical examination, namely separately for normal weight and for overweight pregnant women.

## Methods

### Study population

In this prospective blinded observational study we investigated the accuracy of clinical Leopold’s manoeuvres as compared to ultrasound measurements in fetal weight estimation, with the actual birth weight as the gold standard.

This is a prospective blinded analysis of a cohort of all consecutive women giving birth, including vertex and breech, singleton gestations who presented for labour ≥37 weeks from January 2016 to May 2017 at our department.

To avoid selection bias and perform a real live evaluation, we examined all consecutive women registered for delivery and ultimately delivered at ≥37 weeks. No preterm deliveries prior to 37 weeks are done at our department, but are sent antenatally to a secondary referral centre. Therefore, there are no data on preterm deliveries in our data set.

Cases of both spontaneous labour and induction of labour were included as well as planned (primary) and unplanned (secondary) caesarean sections (see Table 1). No fetal abnormalities were detected in our group of pregnant women.

**Table 1:** Patient characteristics

	*n* = 543	%
Maternal Age	29.2 ± 5.0	
Primiparous	269	49.5
Multiparous	274	50.5
Mean gestational age at examination [Weeks ± SD in days]	37 + 3/7 (262 d) ± 6.8d	
Mean gestational age at time of delivery [weeks ± SD in days]	39 + 2/7 (275 d) ± 8d	
Mean actual birth weight [g]	3382.9 ± 400.2	
Median time estimation to birth [in days ± SD]	15.6 ± 8	
Mode of delivery		
Spontaneous vaginal delivery	342	63.0
Operative vaginal delivery	45	8.3
Caesarean section	156 (100%)	28.7
Planned/Primary	57 (36.5%)	10.5
Unplanned/Secondary (including failed induction of labour)	99 (63.5%)	18.2
Mean maternal BMI [kg/m^2^]	23.9 ± 4.8	
BMI < 25	379	69.8
BMI 25–99	164	30.2
Spontaneous onset of labour	429	79.0
Induced onset of labour	114	21.0
Gestational diabetes	29	5.3
Pre-existing diabetes	2	0.4
Chronic or gestational hypertension	9	1.7
Preeclampsia	13	2.4

The results were documented systematically during and analysed after the study period. All data were analysed in anonymized form. We did not change the pre-existing routine examination.

### Clinical setting and fetal weight estimation by ultrasound and Leopold’s manoeuvres

At our institution the standard of care consists of registering pregnant women for delivery around the 37th week of their pregnancy.

The clinical setting at registration for delivery is as follows: 1.) Patient’s history taken by examining midwife; 2.) Cardiotocography (CTG) for 30 min in pregnant women at risk; 3) basic obstetric vaginal and abdominal examination with Leopold’s manoeuvres by midwife and documentation of EFW (blinded to the physician (sonographer)); 4) ultrasound biometric measurements (GE© E6, 3.5-MHz abdominal transducer) of the fetus by the physician (one of six consultants or one of two residents) including EFW (blinded to the examining midwife) registered in a nationwide electronic documentary system (PIA/Viewpoint© by LB-Systems©); 5) pre-delivery discussion with the physician regarding possible risks and mode of delivery.

Both the midwife and the physician (sonographer) were blinded to the documentation of the weight of prior babies, and pregnant women were asked not to disclose this information to avoid bias.

Every Friday, after the last delivery registration appointment of the week, the measurements were released for comparison. If discrepancies were noticed (> 500 g), these pregnant women were asked to return for re-counselling. Decisions were then based on the ultrasound measurements and their interpretation by a consultant.

Calculation by the ultrasound machine and the PIA/Viewpoint© system is based on Hadlock’s formula [10] including measurement of biparietal diameter (BPD), head circumference (HC), abdominal circumference (AC) and femur length (FL). The results are discussed in a shared decision-making process between the examiner and the mother/parents to plan the mode of delivery.

Fetal weight estimation by midwives using Leopold’s manoeuvres is provided as a point estimate rounded off to the nearest 100 g by the examining midwife.

All examiners, 13 midwives, six consultants and two residents had a level of professional experience of at least 3 years, as both residents were in their fourth and last year of residency. The range of experience among midwives was 5 years to up to 34 years (mean 16.6), among consultants and residents between four and 34 years (mean 11.8).

As previously mentioned, we did not change the pre-existing routine examination, and the midwives already performed Leopold’s manoeuvres as a non-invasive examination for fetal weight estimation before we started our study. The institutional review board (IRB) decision was obtained from the Tauernkliniken GmbH IRB before recruitment for the full trial began in December 2015 (Ref.nr. IRB TK 01_10/2015). All women gave verbal informed consent to participate, which was recorded in the patient’s records.

Maternal demographics as well as pregnancy and neonatal outcome information were extracted from electronic medical records (PIA/Viewpoint© by LB-Systems©).

In order to extrapolate EFW (Leopold and US) from the examination on the date of birth registration to the actual date of birth, we used the complementary percentile curve for the Austrian population (separately available for girls and boys) (Heim et al., unpublished data).

BMI was evaluated for its impact on clinical estimation of fetal weight. Maternal BMI was calculated from height (self-reported) and weight (measured) at the time of admission and was divided into sub-categories of < 25 kg/m^2^ and ≥ 25 kg/m^2^.

Gestational age at registration for delivery was evaluated in intervals of 37 to 39 6/7 weeks, 40 to 40 6/7 weeks, and ≥ 41 weeks.

The outcome was to compare overall absolute error, overall absolute percent error, absolute percent error > 10% and absolute percent error > 20% for weight estimation by ultrasound and by Leopold’s manoeuvres versus the actual birth weight as the given gold standard. The estimations and extrapolations were performed according to validated methods to the best of our knowledge. The median time between estimation and birth is shown in Table 1.

### Statistical analysis

Baseline characteristics of the study cohort were reported using descriptive statistics.

We calculated the mean and standard deviation (SD) of maternal age (years), duration of pregnancy (weeks + 6/7 days), fetal weight at birth (grams), body mass index (BMI) (kg/m^2^), parity, mode of delivery (spontaneous, vaginal operative, Caesarean section), induction of labour and maternal risk factors (gestational diabetes, hypertension, preeclampsia) for univariate descriptive analysis (“patient characteristics”). Absolute errors (equal to the absolute value of the difference between the estimate and the observed weight at birth date) in the estimates were calculated, reporting the mean and SD for the Leopold and the US estimates. It seemed to be practice-relevant to report the proportion of cases with an absolute error ≥ 500 g [20]. Additionally, we report absolute percent errors (mean SD), absolute percent errors > 10% and absolute percent errors > 20%.

To test for differences in the absolute errors and the absolute percent errors between Leopold and ultrasound estimates we used the paired T test. For the proportion of absolute errors ≥500 g, absolute percent error > 10% and absolute percent error > 20% we used the McNemar test statistics for paired samples.

We conducted the above analysis separately for normal weight and for overweight pregnant women.

In order to investigate the effects of BMI on estimate errors, we performed a descriptive analysis as described above, namely separately for the two groups (using two sample tests instead of paired tests). We stratified the results for BMI for < 25 kg/m^2^ (normal weight) and ≥ 25 kg/m^2^ (overweight).

Normality test was applied first by visual inspection of the respective histograms and then formally by applying the Shapiro-Wilk Test.

All statistical analyses were performed using Stata/SE 13.1, Special Edition (College Station, TX, USA).

## Results

### Patient characteristics

A total of 547 pregnant women were eligible to be included, four pregnant women had to be excluded as they gave birth at a different department after registration at our department. Therefore, 543 pregnant women were included in the data analysis. Of the pregnant women in our cohort 5.3% had gestational diabetes. Due to mandatory gestational diabetes screening during pregnancy in Austria and a close follow-up after registration for delivery at our department, which may represent a situation different from that in other countries, we are able to state that these 5.3% pregnant women with gestational diabetes in our cohort were exactly monitored with blood sugar testing. Due to normal results in all 29 patients, who had either diet or insulin, the pregnancies with gestational diabetes in our cohort were comparable to normal pregnancies.

Patient characteristics can be found in Table 1.

Fetal weight estimation: ultrasound versus Leopold’s manoeuvres.

No statistically significant difference was seen in the accuracy of fetal weight estimation performed with Leopold’s manoeuvres versus ultrasound in any absolute error calculations of normal weight women giving birth. This can be seen from Table 2 at the time of delivery registration.

**Table 2:** Accuracy of both weight estimations regarding effective birth weight in all normal weight pregnant women

EFW	Leopold’s manoeuvres	Ultrasound	*p* value
Absolute error [g]	279 ± 225	257 ± 204	0.0696^a^
Absolute error > 500g [%]	17.2	12.9	0.0805^b^
Absolute % error [g]	8.6 ± 7.5	7.9 ± 6.5	0.051^a^
Absolute % error > 10% [%]	33.5	29.6	0.155^b^
Absolute % error > 20% [%]	7.1	6.9	1.0^b^

^a^Paired T test, ^b^Exact McNemar test

A statistically significant difference in the accuracy of fetal weight estimation was observed in favour of ultrasound in all absolute error calculations performed in overweight women giving birth. This can be seen from Table 3 at the time of delivery registration.

**Table 3:** Accuracy of both weight estimations regarding actual birth weight in all overweight pregnant women

EFW	Leopold’s manoeuvres	Ultrasound	*p* value
Absolute error [g]	343 ± 250	245 ± 190	<0.001^a^
Absolute error > 500g [%]	22.6	9.1	0.0002^b^
Absolute % error [g]	10.1 ± 7.8	7.3 ± 6.1	<0.001^a^
Absolute % error > 10% [%]	42.1	24.4	0.0002^b^
Absolute % error > 20% [%]	12.8	4.3	0.0026^b^

^a^Paired T test, ^b^Exact McNemar test

A statistically significant difference in the accuracy of fetal weight estimation was observed in favour of ultrasound in all absolute error calculations performed in all women giving birth. This can be seen from Table 4 at the time of delivery registration.

**Table 4:** Accuracy of both weight estimations regarding actual birth weight in all pregnant women

EFW	Leopold’s manoeuvres	Ultrasound	*p* value
Absolute error [g]	298 ± 235	254 ± 200	<0.001^a^
Absolute error > 500g [%]	18.8	11.8	0.0003^b^
Absolute % error [g]	9.1 ± 7.6	7.7 ± 6.4	<0.001^a^
Absolute % error > 10% [%]	36.1	28.0	0.0004^b^
Absolute % error > 20% [%]	8.8	6.1	0.036^b^

^a^Paired T test, ^b^Exact McNemar test

**Fig. 1:**
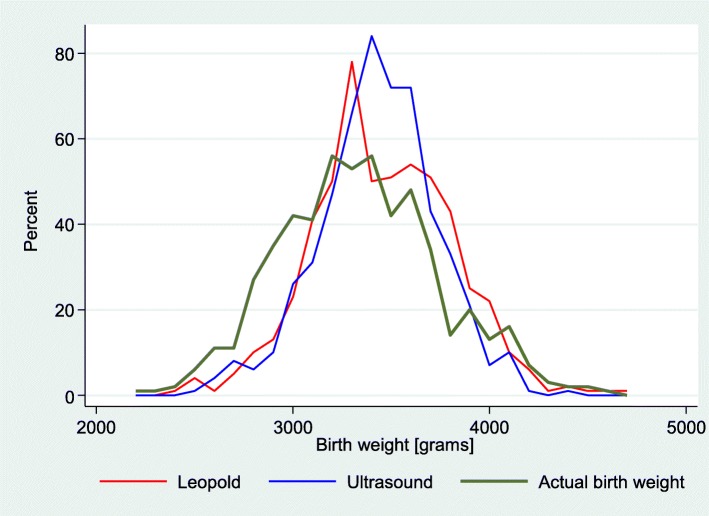
Estimated fetal weight from the time of examination in relation to the actual birth weight

Density of distribution of estimated fetal weight as compared to actual birthweight established by ultrasound versus palpation is shown in Fig. 1. The data in the present study show that the estimates made by the examiners, whether physicians or midwives, whether with ultrasound or clinical palpation, were close together in normal weight women.

**Fig. 2:**
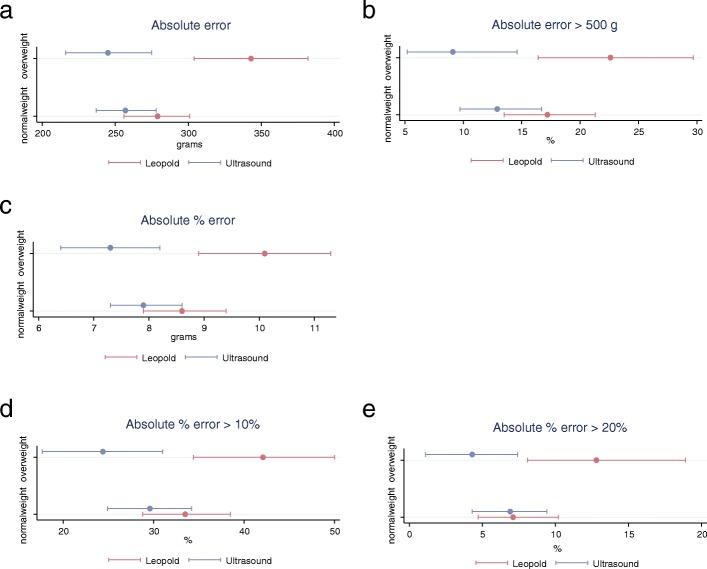
**a**) Absolute error of estimated fetal weight (± 95% CI), **b**) Absolute error > 500 g of estimated fetal weight (± 95% CI), **c**) Absolute % error of estimated fetal weight (± 95% CI), **d**) Absolute % error > 10 % of estimated fetal weight (± 95% CI), **e**) Absolute % error > 20% of estimated fetal weight (± 95% CI) in normal weight and overweight women with Leopold´s maneuvers or ultrasound

Fetal weight estimations of normal weight and overweight women with either Leopold’s manoeuvres or ultrasound displaying absolute error, absolute error > 500 g, absolute percent error, absolute error > 10% and absolute error > 20%, including 95% confidence intervals (± 95% CI) are shown in detail in Fig. 2a-e.

## Discussion

### Main findings

In this prospective blinded observational study we found a statistically significant difference in the accuracy of fetal weight estimation in favour of ultrasound in all absolute error calculations performed in overweight women giving birth, however no statistically significant difference in normal weight women giving birth.

With regard to the mode of delivery and exact timing in the event that it is necessary to induce labour, the accuracy of fetal weight estimation is of key importance in the obstetrician’s decision-making process shared with the expectant mother and has been a matter of discussion for many years [21].

A deviation of 500 g could have a significant impact on the shared decision-making process, particularly with regard to cut-off levels given in international guidelines [20, 21].

### Interpretation in light of other evidence

First, our study demonstrates a statistically significant difference in the accuracy of fetal weight estimation in favour of ultrasound in all absolute error calculations made in overweight women giving birth. With regard to an absolute error > 500 g that is clinically relevant for the obstetric decision-making process, a significant difference was evident between the two methods when used in overweight pregnant women.

These results are in line with previously published data [22–24].

Second, no statistically significant difference was seen in the accuracy of fetal weight estimation obtained with Leopold’s manoeuvres versus ultrasound in absolute error calculations performed in normal weight women giving birth.

The most established way to estimate fetal weight is the ultrasound method, as previously described [10–12] and most commonly performed with three measurements fitted into an algorithm designed by Hadlock et al. [10]. Other approaches like MRI or soft-tissue measurements have proved to not be of added benefit [13, 14].

International percentile curves for EFW, calculated after studies of fetuses in Anglo-Saxon countries and used to check the week-adapted weight of the unborn fetuses worldwide, may not be the right strategy because they pursue a one-size-fits-all policy in approaching what is too large or too small [25, 26].

Very recently Nicolaides et al. [27] published a study aiming to develop fetal and neonatal population weight charts. The rationale was that reference ranges of EFW are representative for the whole population, while the traditional approach of deriving birth-weight (BW) charts is misleading, because a large proportion of babies born preterm arises from pathological pregnancy [27]. The study qualified that the desire for a single international standard for all countries is not appropriate [27]. This has been demonstrated in different studies before by likely differences in percentile curves as a consequence of underlying differences in the study populations [28, 29].

The long-standing, mainly midwifery-based tradition of clinical weight estimation by means of Leopold’s manoeuvres is a non-invasive approach to fetal weight estimation that is used when ultrasound is not available [1, 15].

The conclusions published in the literature regarding fetal weight estimation are inconsistent, with some studies favouring ultrasound measurement [30–34]. Goetzinger et al. reported a lack of accuracy in fetal weight estimation when using Leopold’s manoeuvres [35]. Several prospective studies were able to show advantages of clinical palpation like Leopold’s manoeuvres in predicting fetal macrosomia [14, 26, 36, 37], and the accuracy of fetal weight estimation when using ultrasound biometry has been shown to be no better than that of Leopold’s manoeuvres [21]. Still other studies report an advantage for them for fetal weight estimation [38–44].

High incongruence of study designs and the difference in approaches regarding time of examination, extrapolation of absolute error in matters of actual birth weight, examiner’s experience etc. might explain the variety of different results obtained.

As several studies have shown body mass index (BMI) to affect the accuracy of EFW [16–19], we decided to stratify between normal weight and overweight pregnant women in our cohort.

We found a significant difference between ultrasound and clinical palpation with Leopold’s manoeuvres regarding fetal weight estimation in overweight pregnant women.

The difficulty of obstetric ultrasound examinations mostly grows with increasing maternal BMI due to diminished visualization, but its impact on fetal weight estimation is described controversially in the existing literature [19, 22, 23].

Furthermore, we found no significant difference between clinical palpation with Leopold’s manoeuvres and ultrasound for fetal weight estimation in normal weight pregnant women.

Our study included women who gave birth within the mean time of 13 days after fetal weight estimation. Some study data and systematic review results show that the most accurate estimates can be expected between four and 7 days before delivery [45–47]. Nevertheless, we did not change our management procedures and estimated fetal weight pragmatically at registration for delivery. Furthermore, fetal weight estimation at term is suspected of doing more harm than good and is the matter of ongoing peer discussion very recently [48].

In this connection we used the most recent Austrian percentile curves for the expected actual birth weight (Heim et al., in submission) and compared them with the actual birth weight to validate the measurement results. Several studies previously showed that Hadlock’s formula is the most consistently used for normal clinical cohorts [12, 49].

### Strengths and limitations

The strengths of our study are that we present prospective data resulting from a pragmatic assessment of pregnant women as they arrived at our department for delivery, representing a normal life situation. Furthermore, the stratification of normal weight and overweight women regarding the effect of BMI on the accuracy of EFW is the major contribution of our study to the existing literature. In the analysis we included all consecutive women giving birth at our department, thus avoiding a selection bias.

A limitation of our study is the borderline significance of errors in the group of normal weight pregnant women, which might become significant for a larger pool of patients.

We did not investigate inter-observer variation. The time interval between estimation and delivery represents the real life clinical setting at our department.

## Conclusions

The data obtained in our prospective blinded observational study show ultrasound to have a significantly better accuracy in fetal weight estimation in overweight pregnant women than Leopold’s manoeuvres. However, no statistically significant difference between the two methods was observed in normal weight pregnant women.

The clinical method using Leopold’s manoeuvres might be useful in countries with poor infrastructure and thus poor availability of ultrasound devices.

## Abbreviations

AC: Abdominal circumference
AC: Abdominal circumference
BMI: Body mass index
BPD: Biparietal diameter
BW: Birth weight
CI: Confidence intervals
CTG: Cardiotocography
EFW: Estimated fetal weight
FL: Femur length
g: grams
HC: Head circumference
IRB: Institutional review board
SD: Standard deviation
TK: Tauernkliniken
